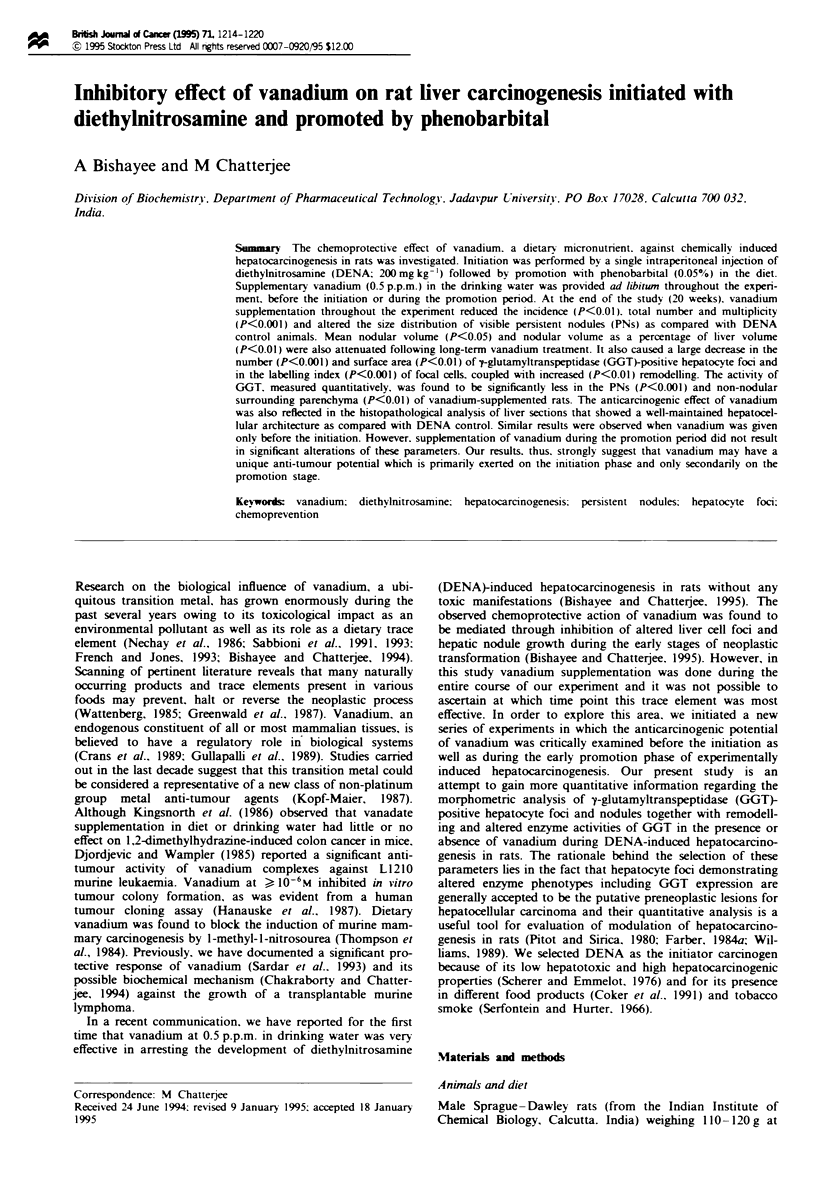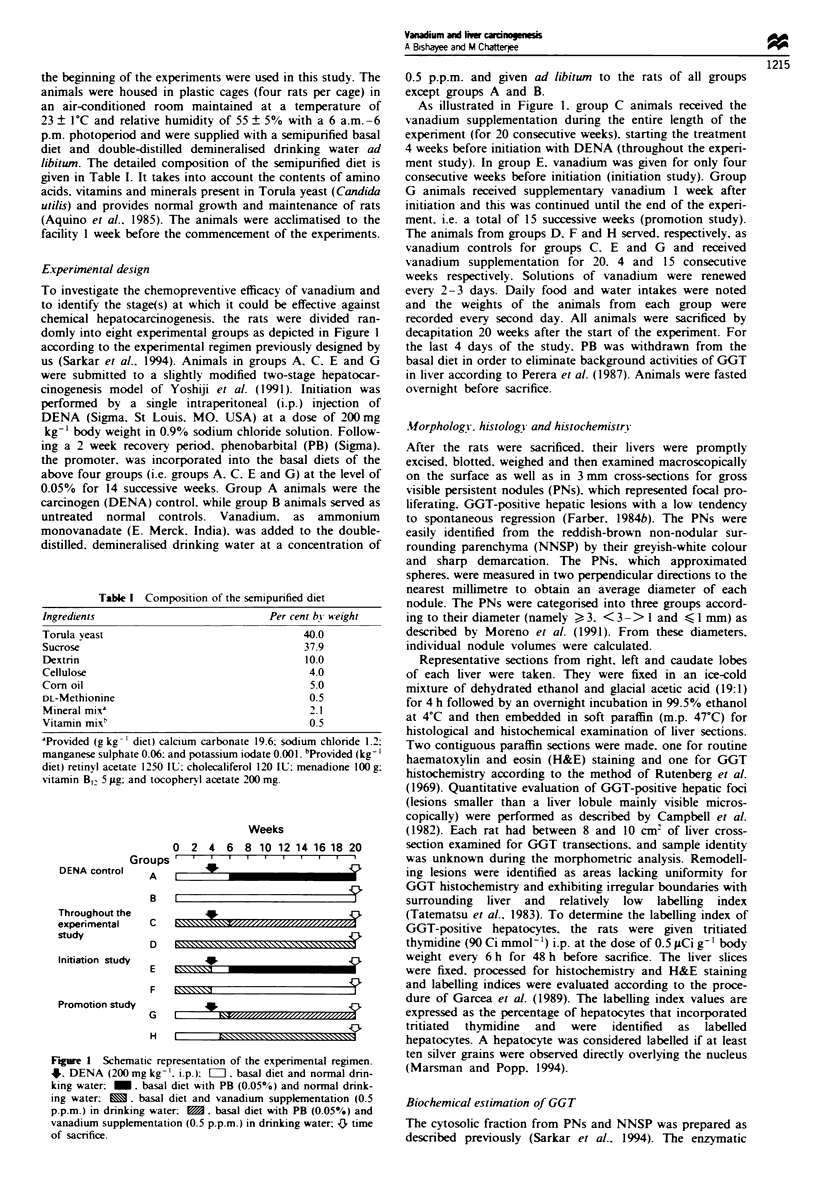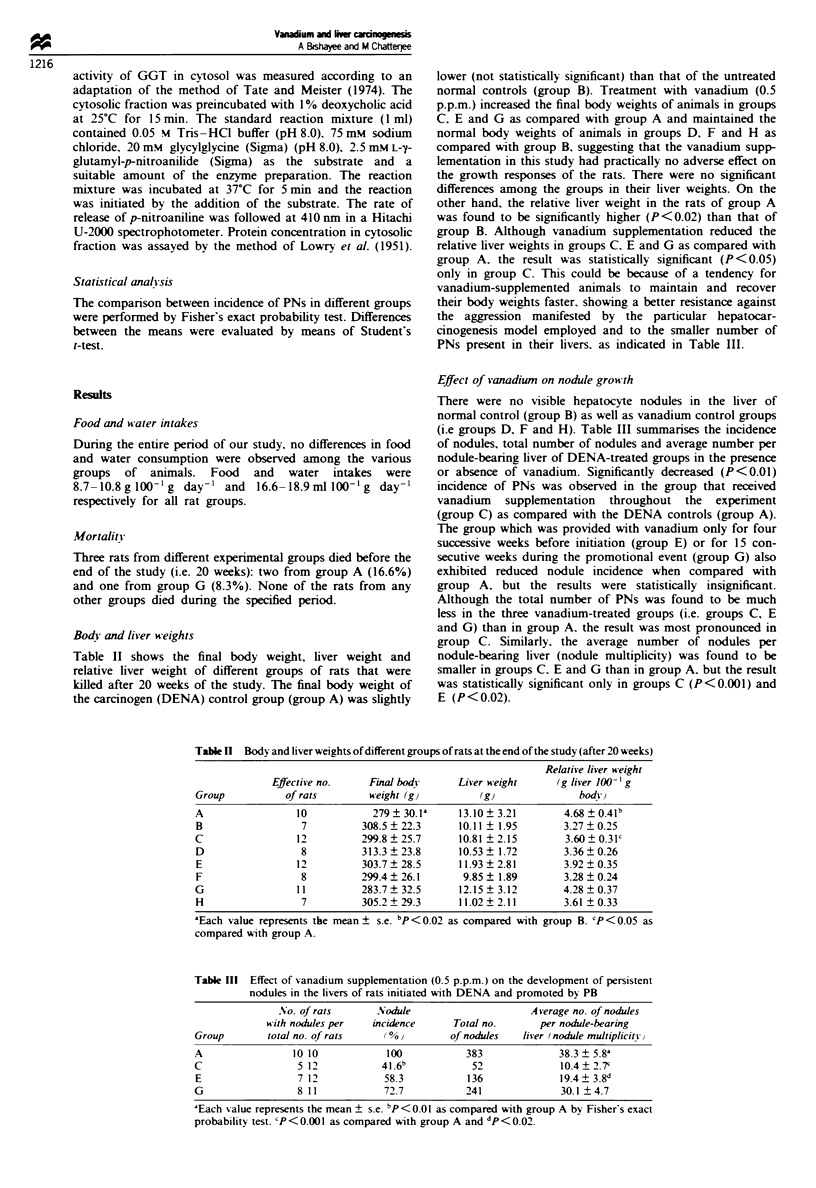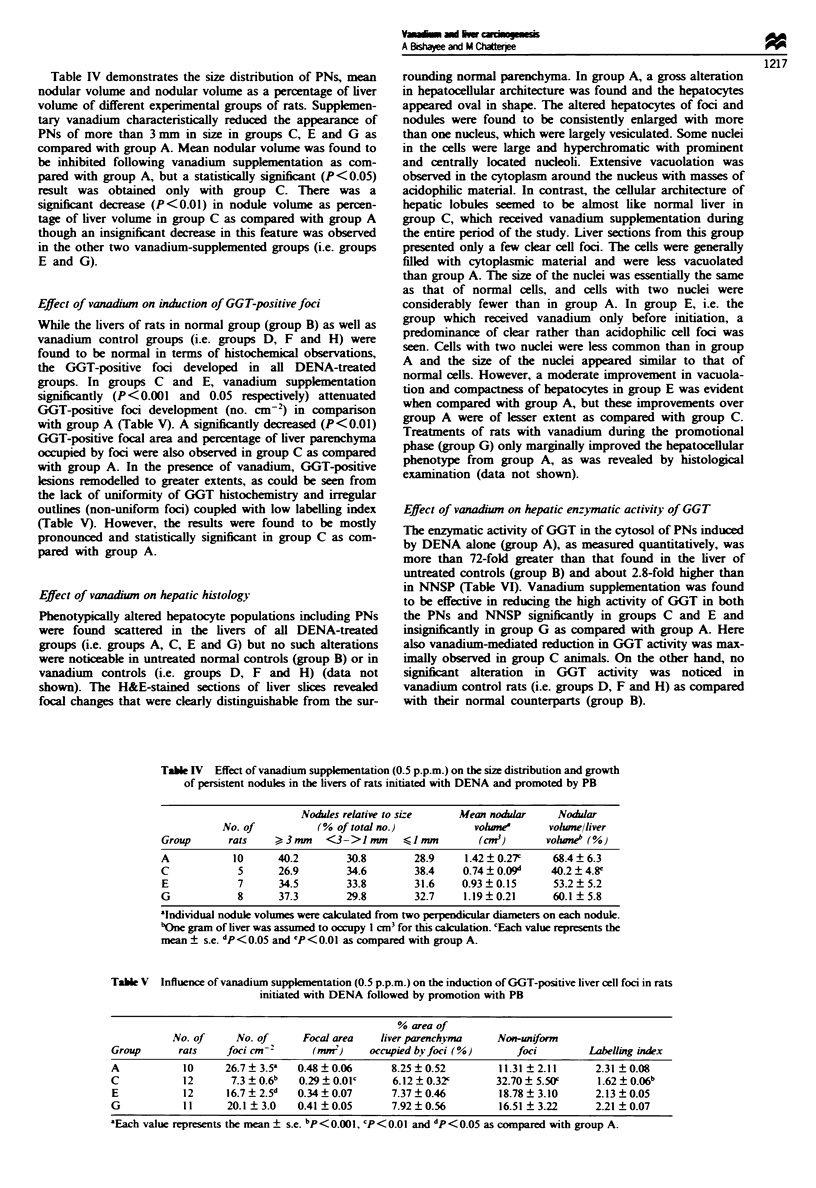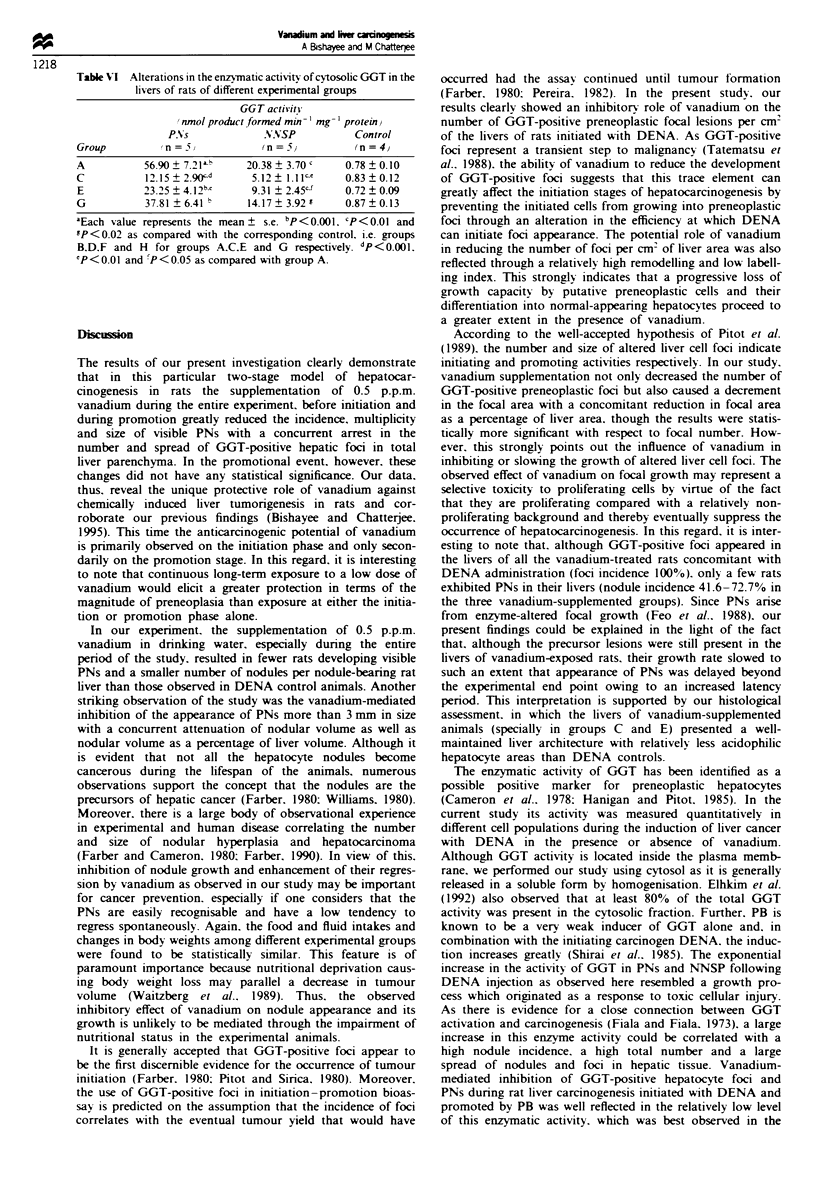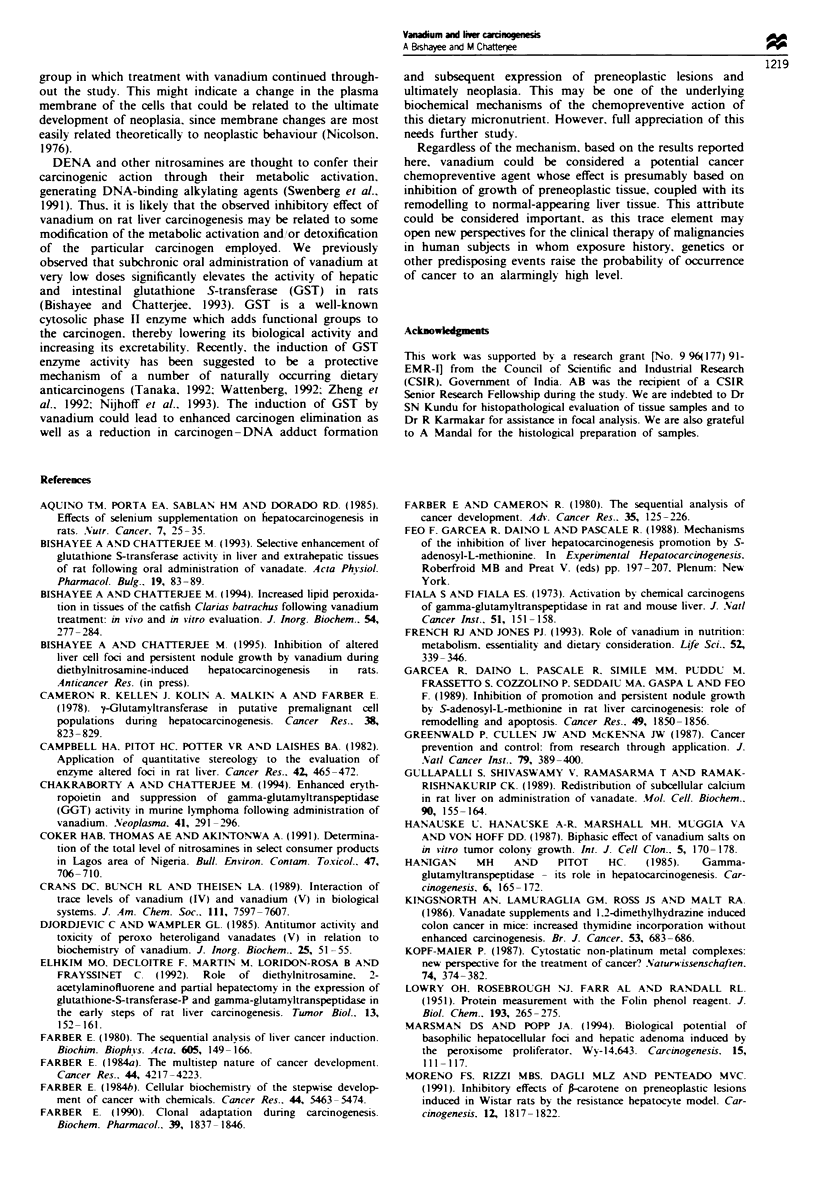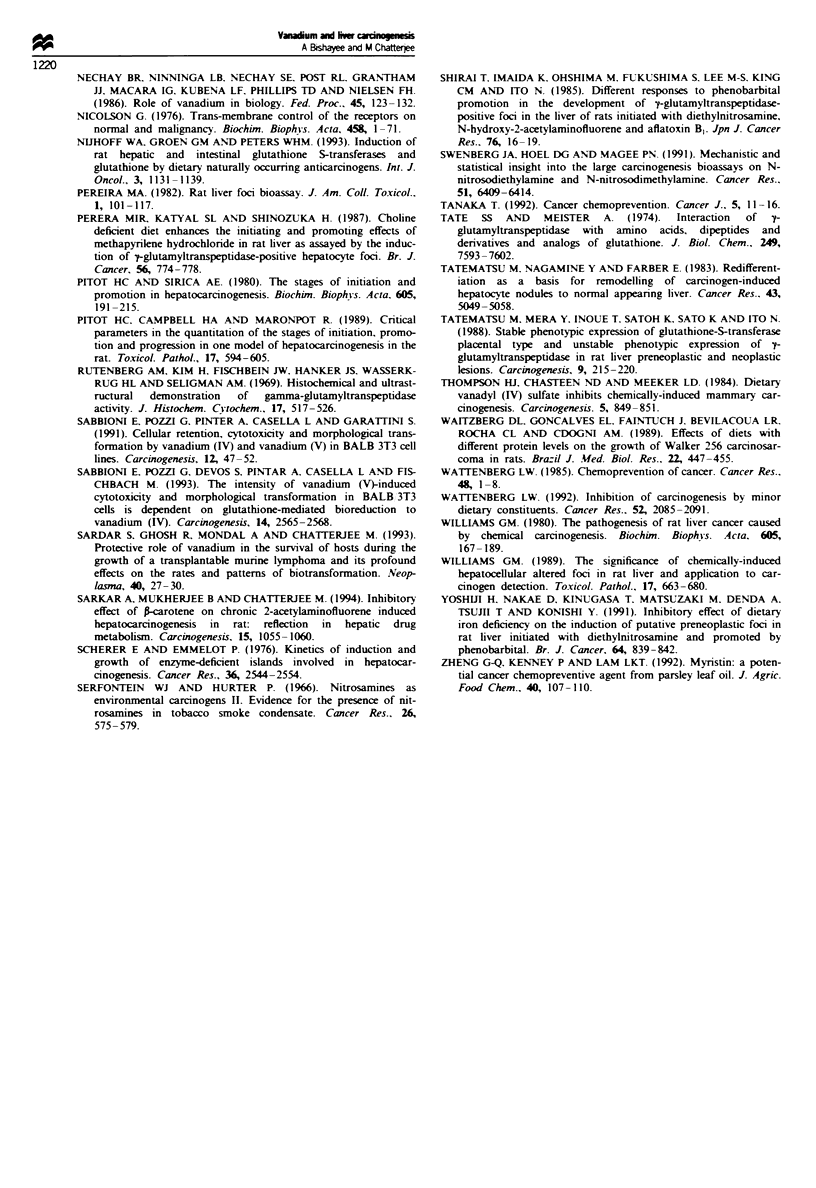# Inhibitory effect of vanadium on rat liver carcinogenesis initiated with diethylnitrosamine and promoted by phenobarbital.

**DOI:** 10.1038/bjc.1995.236

**Published:** 1995-06

**Authors:** A. Bishayee, M. Chatterjee

**Affiliations:** Department of Pharmaceutical Technology, Jadavpur University, Calcutta, India.

## Abstract

The chemoprotective effect of vanadium, a dietary micronutrient, against chemically induced hepatocarcinogenesis in rats was investigated. Initiation was performed by a single intraperitoneal injection of diethylnitrosamine (DENA; 200 mg kg-1) followed by promotion with phenobarbital (0.05%) in the diet. Supplementary vanadium (0.5 p.p.m.) in the drinking water was provided ad libitum throughout the experiment, before the initiation or during the promotion period. At the end of the study (20 weeks), vanadium supplementation throughout the experiment reduced the incidence (P < 0.01), total number and multiplicity (P < 0.001) and altered the size distribution of visible persistent nodules (PNs) as compared with DENA control animals. Mean nodular volume (P < 0.05) and nodular volume as a percentage of liver volume (P < 0.01) were also attenuated following long-term vanadium treatment. It also caused a large decrease in the number (P < 0.001) and surface area (P < 0.01) of gamma-glutamyltranspeptidase (GGT)-positive hepatocyte foci and in the labelling index (P < 0.001) of focal cells, coupled with increased (P < 0.01) remodelling. The activity of GGT, measured quantitatively, was found to be significantly less in the PNs (P < 0.001) and non-nodular surrounding parenchyma (P < 0.01) of vanadium-supplemented rats. The anticarcinogenic effect of vanadium was also reflected in the histopathological analysis of liver sections that showed a well-maintained hepatocellular architecture as compared with DENA control. Similar results were observed when vanadium was given only before the initiation. However, supplementation of vanadium during the promotion period did not result in significant alterations of these parameters. Our results, thus, strongly suggest that vanadium may have a unique anti-tumour potential which is primarily exerted on the initiation phase and only secondarily on the promotion stage.


British Journal of Cancer (1995) 71, 1214-1220

© 1995 Stockton Press Ltd All rights reserved 0007-0920/95 $12.00

### Inhibitory effect of vanadium on rat liver carcinogenesis initiated with diethylnitrosamine and promoted by phenobarbital

A Bishayee and M Chatterjee

*Division of Biochemistry, Department of Pharmaceutical Technology, Jadavpur University, PO Box 17028, Calcutta 700 032, India.*

**Summary** The chemoprotective effect of vanadium, a dietary micronutrient, against chemically induced hepatocarcinogenesis in rats was investigated. Initiation was performed by a single intraperitoneal injection of diethylnitrosamine (DENA; 200 mg $kg^{-1}$) followed by promotion with phenobarbital (0.05%) in the diet. Supplementary vanadium (0.5 p.p.m.) in the drinking water was provided *ad libitum* throughout the experiment, before the initiation or during the promotion period. At the end of the study (20 weeks), vanadium supplementation throughout the experiment reduced the incidence ($P<0.01$), total number and multiplicity ($P<0.001$) and altered the size distribution of visible persistent nodules (PNs) as compared with DENA control animals. Mean nodular volume ($P<0.05$) and nodular volume as a percentage of liver volume ($P<0.01$) were also attenuated following long-term vanadium treatment. It also caused a large decrease in the number ($P<0.001$) and surface area ($P<0.01$) of γ-glutamyltranspeptidase (GGT)-positive hepatocyte foci and in the labelling index ($P<0.001$) of focal cells, coupled with increased ($P<0.01$) remodelling. The activity of GGT, measured quantitatively, was found to be significantly less in the PNs ($P<0.001$) and non-nodular surrounding parenchyma ($P<0.01$) of vanadium-supplemented rats. The anticarcinogenic effect of vanadium was also reflected in the histopathological analysis of liver sections that showed a well-maintained hepatocellular architecture as compared with DENA control. Similar results were observed when vanadium was given only before the initiation. However, supplementation of vanadium during the promotion period did not result in significant alterations of these parameters. Our results, thus, strongly suggest that vanadium may have a unique anti-tumour potential which is primarily exerted on the initiation phase and only secondarily on the promotion stage.

**Keywords:** vanadium; diethylnitrosamine; hepatocarcinogenesis; persistent nodules; hepatocyte foci; chemoprevention

Research on the biological influence of vanadium, a ubiquitous transition metal, has grown enormously during the past several years owing to its toxicological impact as an environmental pollutant as well as its role as a dietary trace element (Nechay *et al.*, 1986; Sabbioni *et al.*, 1991, 1993; French and Jones, 1993; Bishayee and Chatterjee, 1994). Scanning of pertinent literature reveals that many naturally occurring products and trace elements present in various foods may prevent, halt or reverse the neoplastic process (Wattenberg, 1985; Greenwald *et al.*, 1987). Vanadium, an endogenous constituent of all or most mammalian tissues, is believed to have a regulatory role in biological systems (Crans *et al.*, 1989; Gullapalli *et al.*, 1989). Studies carried out in the last decade suggest that this transition metal could be considered a representative of a new class of non-platinum group metal anti-tumour agents (Kopf-Maier, 1987). Although Kingsnorth *et al.* (1986) observed that vanadate supplementation in diet or drinking water had little or no effect on 1,2-dimethylhydrazine-induced colon cancer in mice, Djordjevic and Wampler (1985) reported a significant anti-tumour activity of vanadium complexes against L1210 murine leukaemia. Vanadium at $\geqslant 10^{-6}$ M inhibited *in vitro* tumour colony formation, as was evident from a human tumour cloning assay (Hanauske *et al.*, 1987). Dietary vanadium was found to block the induction of murine mammary carcinogenesis by 1-methyl-1-nitrosourea (Thompson *et al.*, 1984). Previously, we have documented a significant protective response of vanadium (Sardar *et al.*, 1993) and its possible biochemical mechanism (Chakraborty and Chatterjee, 1994) against the growth of a transplantable murine lymphoma.

In a recent communication, we have reported for the first time that vanadium at 0.5 p.p.m. in drinking water was very effective in arresting the development of diethylnitrosamine (DENA)-induced hepatocarcinogenesis in rats without any toxic manifestations (Bishayee and Chatterjee, 1995). The observed chemoprotective action of vanadium was found to be mediated through inhibition of altered liver cell foci and hepatic nodule growth during the early stages of neoplastic transformation (Bishayee and Chatterjee, 1995). However, in this study vanadium supplementation was done during the entire course of our experiment and it was not possible to ascertain at which time point this trace element was most effective. In order to explore this area, we initiated a new series of experiments in which the anticarcinogenic potential of vanadium was critically examined before the initiation as well as during the early promotion phase of experimentally induced hepatocarcinogenesis. Our present study is an attempt to gain more quantitative information regarding the morphometric analysis of γ-glutamyltranspeptidase (GGT)-positive hepatocyte foci and nodules together with remodelling and altered enzyme activities of GGT in the presence or absence of vanadium during DENA-induced hepatocarcinogenesis in rats. The rationale behind the selection of these parameters lies in the fact that hepatocyte foci demonstrating altered enzyme phenotypes including GGT expression are generally accepted to be the putative preneoplastic lesions for hepatocellular carcinoma and their quantitative analysis is a useful tool for evaluation of modulation of hepatocarcinogenesis in rats (Pitot and Sirica, 1980; Farber, 1984*a*; Williams, 1989). We selected DENA as the initiator carcinogen because of its low hepatotoxic and high hepatocarcinogenic properties (Scherer and Emmelot, 1976) and for its presence in different food products (Coker *et al.*, 1991) and tobacco smoke (Serfontein and Hurter, 1966).

### Materials and methods

### *Animals and diet*

Male Sprague-Dawley rats (from the Indian Institute of Chemical Biology, Calcutta, India) weighing 110-120 g at

Correspondence: M Chatterjee
Received 24 June 1994; revised 9 January 1995; accepted 18 January 1995

the beginning of the experiments were used in this study. The animals were housed in plastic cages (four rats per cage) in an air-conditioned room maintained at a temperature of 23 ± 1°C and relative humidity of 55 ± 5% with a 6 a.m.–6 p.m. photoperiod and were supplied with a semipurified basal diet and double-distilled demineralised drinking water *ad libitum*. The detailed composition of the semipurified diet is given in Table I. It takes into account the contents of amino acids, vitamins and minerals present in Torula yeast (*Candida utilis*) and provides normal growth and maintenance of rats (Aquino *et al*., 1985). The animals were acclimatised to the facility 1 week before the commencement of the experiments.

### *Experimental design*

To investigate the chemopreventive efficacy of vanadium and to identify the stage(s) at which it could be effective against chemical hepatocarcinogenesis, the rats were divided randomly into eight experimental groups as depicted in Figure 1 according to the experimental regimen previously designed by us (Sarkar *et al*., 1994). Animals in groups A, C, E and G were submitted to a slightly modified two-stage hepatocarcinogenesis model of Yoshiji *et al*. (1991). Initiation was performed by a single intraperitoneal (i.p.) injection of DENA (Sigma, St Louis, MO, USA) at a dose of 200 mg kg$^{-1}$ body weight in 0.9% sodium chloride solution. Following a 2 week recovery period, phenobarbital (PB) (Sigma), the promoter, was incorporated into the basal diets of the above four groups (i.e. groups A, C, E and G) at the level of 0.05% for 14 successive weeks. Group A animals were the carcinogen (DENA) control, while group B animals served as untreated normal controls. Vanadium, as ammonium monovanadate (E. Merck, India), was added to the double-distilled, demineralised drinking water at a concentration of 0.5 p.p.m. and given *ad libitum* to the rats of all groups except groups A and B.

As illustrated in Figure 1, group C animals received the vanadium supplementation during the entire length of the experiment (for 20 consecutive weeks), starting the treatment 4 weeks before initiation with DENA (throughout the experiment study). In group E, vanadium was given for only four consecutive weeks before initiation (initiation study). Group G animals received supplementary vanadium 1 week after initiation and this was continued until the end of the experiment, i.e. a total of 15 successive weeks (promotion study). The animals from groups D, F and H served, respectively, as vanadium controls for groups C, E and G and received vanadium supplementation for 20, 4 and 15 consecutive weeks respectively. Solutions of vanadium were renewed every 2–3 days. Daily food and water intakes were noted and the weights of the animals from each group were recorded every second day. All animals were sacrificed by decapitation 20 weeks after the start of the experiment. For the last 4 days of the study, PB was withdrawn from the basal diet in order to eliminate background activities of GGT in liver according to Perera *et al*. (1987). Animals were fasted overnight before sacrifice.

**Table I** Composition of the semipurified diet

| *Ingredients* | *Per cent by weight* |
|---|---|
| Torula yeast | 40.0 |
| Sucrose | 37.9 |
| Dextrin | 10.0 |
| Cellulose | 4.0 |
| Corn oil | 5.0 |
| DL-Methionine | 0.5 |
| Mineral mix[a] | 2.1 |
| Vitamin mix[b] | 0.5 |

[a]Provided (g kg$^{-1}$ diet) calcium carbonate 19.6; sodium chloride 1.2; manganese sulphate 0.06; and potassium iodate 0.001. [b]Provided (kg$^{-1}$ diet) retinyl acetate 1250 IU; cholecaliferol 120 IU; menadione 100 g; vitamin $B_{12}$ 5 μg; and tocopheryl acetate 200 mg.

**Figure 1** Schematic representation of the experimental regimen. ⬇, DENA (200 mg kg$^{-1}$, i.p.); ☐, basal diet and normal drinking water; ■, basal diet with PB (0.05%) and normal drinking water; ▧, basal diet and vanadium supplementation (0.5 p.p.m.) in drinking water; ▨, basal diet with PB (0.05%) and vanadium supplementation (0.5 p.p.m.) in drinking water; ⇩ time of sacrifice.

### *Morphology, histology and histochemistry*

After the rats were sacrificed, their livers were promptly excised, blotted, weighed and then examined macroscopically on the surface as well as in 3 mm cross-sections for gross visible persistent nodules (PNs), which represented focal proliferating, GGT-positive hepatic lesions with a low tendency to spontaneous regression (Farber, 1984*b*). The PNs were easily identified from the reddish-brown non-nodular surrounding parenchyma (NNSP) by their greyish-white colour and sharp demarcation. The PNs, which approximated spheres, were measured in two perpendicular directions to the nearest millimetre to obtain an average diameter of each nodule. The PNs were categorised into three groups according to their diameter (namely ⩾3, <3–>1 and ⩽1 mm) as described by Moreno *et al*. (1991). From these diameters, individual nodule volumes were calculated.

Representative sections from right, left and caudate lobes of each liver were taken. They were fixed in an ice-cold mixture of dehydrated ethanol and glacial acetic acid (19:1) for 4 h followed by an overnight incubation in 99.5% ethanol at 4°C and then embedded in soft paraffin (m.p. 47°C) for histological and histochemical examination of liver sections. Two contiguous paraffin sections were made, one for routine haematoxylin and eosin (H&E) staining and one for GGT histochemistry according to the method of Rutenberg *et al*. (1969). Quantitative evaluation of GGT-positive hepatic foci (lesions smaller than a liver lobule mainly visible microscopically) were performed as described by Campbell *et al*. (1982). Each rat had between 8 and 10 cm$^2$ of liver cross-section examined for GGT transections, and sample identity was unknown during the morphometric analysis. Remodelling lesions were identified as areas lacking uniformity for GGT histochemistry and exhibiting irregular boundaries with surrounding liver and relatively low labelling index (Tatematsu *et al*., 1983). To determine the labelling index of GGT-positive hepatocytes, the rats were given tritiated thymidine (90 Ci mmol$^{-1}$) i.p. at the dose of 0.5 μCi g$^{-1}$ body weight every 6 h for 48 h before sacrifice. The liver slices were fixed, processed for histochemistry and H&E staining and labelling indices were evaluated according to the procedure of Garcea *et al*. (1989). The labelling index values are expressed as the percentage of hepatocytes that incorporated tritiated thymidine and were identified as labelled hepatocytes. A hepatocyte was considered labelled if at least ten silver grains were observed directly overlying the nucleus (Marsman and Popp, 1994).

### *Biochemical estimation of GGT*

The cytosolic fraction from PNs and NNSP was prepared as described previously (Sarkar *et al*., 1994). The enzymatic activity of GGT in cytosol was measured according to an adaptation of the method of Tate and Meister (1974). The cytosolic fraction was preincubated with 1% deoxycholic acid at 25°C for 15 min. The standard reaction mixture (1 ml) contained 0.05 M Tris–HCl buffer (pH 8.0), 75 mM sodium chloride, 20 mM glycylglycine (Sigma) (pH 8.0), 2.5 mM L-γ-glutamyl-*p*-nitroanilide (Sigma) as the substrate and a suitable amount of the enzyme preparation. The reaction mixture was incubated at 37°C for 5 min and the reaction was initiated by the addition of the substrate. The rate of release of *p*-nitroaniline was followed at 410 nm in a Hitachi U-2000 spectrophotometer. Protein concentration in cytosolic fraction was assayed by the method of Lowry *et al.* (1951).

### *Statistical analysis*

The comparison between incidence of PNs in different groups were performed by Fisher's exact probability test. Differences between the means were evaluated by means of Student's *t*-test.

### Results

### *Food and water intakes*

During the entire period of our study, no differences in food and water consumption were observed among the various groups of animals. Food and water intakes were 8.7–10.8 g $100^{-1}$ g $day^{-1}$ and 16.6–18.9 ml $100^{-1}$ g $day^{-1}$ respectively for all rat groups.

### *Mortality*

Three rats from different experimental groups died before the end of the study (i.e. 20 weeks): two from group A (16.6%) and one from group G (8.3%). None of the rats from any other groups died during the specified period.

### *Body and liver weights*

Table II shows the final body weight, liver weight and relative liver weight of different groups of rats that were killed after 20 weeks of the study. The final body weight of the carcinogen (DENA) control group (group A) was slightly lower (not statistically significant) than that of the untreated normal controls (group B). Treatment with vanadium (0.5 p.p.m.) increased the final body weights of animals in groups C, E and G as compared with group A and maintained the normal body weights of animals in groups D, F and H as compared with group B, suggesting that the vanadium supplementation in this study had practically no adverse effect on the growth responses of the rats. There were no significant differences among the groups in their liver weights. On the other hand, the relative liver weight in the rats of group A was found to be significantly higher ($P<0.02$) than that of group B. Although vanadium supplementation reduced the relative liver weights in groups C, E and G as compared with group A, the result was statistically significant ($P<0.05$) only in group C. This could be because of a tendency for vanadium-supplemented animals to maintain and recover their body weights faster, showing a better resistance against the aggression manifested by the particular hepatocarcinogenesis model employed and to the smaller number of PNs present in their livers, as indicated in Table III.

### *Effect of vanadium on nodule growth*

There were no visible hepatocyte nodules in the liver of normal control (group B) as well as vanadium control groups (i.e groups D, F and H). Table III summarises the incidence of nodules, total number of nodules and average number per nodule-bearing liver of DENA-treated groups in the presence or absence of vanadium. Significantly decreased ($P<0.01$) incidence of PNs was observed in the group that received vanadium supplementation throughout the experiment (group C) as compared with the DENA controls (group A). The group which was provided with vanadium only for four successive weeks before initiation (group E) or for 15 consecutive weeks during the promotional event (group G) also exhibited reduced nodule incidence when compared with group A, but the results were statistically insignificant. Although the total number of PNs was found to be much less in the three vanadium-treated groups (i.e. groups C, E and G) than in group A, the result was most pronounced in group C. Similarly, the average number of nodules per nodule-bearing liver (nodule multiplicity) was found to be smaller in groups C, E and G than in group A, but the result was statistically significant only in groups C ($P<0.001$) and E ($P<0.02$).

**Table II** Body and liver weights of different groups of rats at the end of the study (after 20 weeks)

| *Group* | *Effective no. of rats* | *Final body weight (g)* | *Liver weight (g)* | *Relative liver weight (g liver $100^{-1}$ g body)* |
|---|---|---|---|---|
| A | 10 | 279 ± 30.1[a] | 13.10 ± 3.21 | 4.68 ± 0.41[b] |
| B | 7 | 308.5 ± 22.3 | 10.11 ± 1.95 | 3.27 ± 0.25 |
| C | 12 | 299.8 ± 25.7 | 10.81 ± 2.15 | 3.60 ± 0.31[c] |
| D | 8 | 313.3 ± 23.8 | 10.53 ± 1.72 | 3.36 ± 0.26 |
| E | 12 | 303.7 ± 28.5 | 11.93 ± 2.81 | 3.92 ± 0.35 |
| F | 8 | 299.4 ± 26.1 | 9.85 ± 1.89 | 3.28 ± 0.24 |
| G | 11 | 283.7 ± 32.5 | 12.15 ± 3.12 | 4.28 ± 0.37 |
| H | 7 | 305.2 ± 29.3 | 11.02 ± 2.11 | 3.61 ± 0.33 |

[a]Each value represents the mean ± s.e. [b]$P<0.02$ as compared with group B. [c]$P<0.05$ as compared with group A.

**Table III** Effect of vanadium supplementation (0.5 p.p.m.) on the development of persistent nodules in the livers of rats initiated with DENA and promoted by PB

| *Group* | *No. of rats with nodules per total no. of rats* | *Nodule incidence (%)* | *Total no. of nodules* | *Average no. of nodules per nodule-bearing liver (nodule multiplicity)* |
|---|---|---|---|---|
| A | 10/10 | 100 | 383 | 38.3 ± 5.8[a] |
| C | 5/12 | 41.6[b] | 52 | 10.4 ± 2.7[c] |
| E | 7/12 | 58.3 | 136 | 19.4 ± 3.8[d] |
| G | 8/11 | 72.7 | 241 | 30.1 ± 4.7 |

[a]Each value represents the mean ± s.e. [b]$P<0.01$ as compared with group A by Fisher's exact probability test. [c]$P<0.001$ as compared with group A and [d]$P<0.02$.

Table IV demonstrates the size distribution of PNs, mean nodular volume and nodular volume as a percentage of liver volume of different experimental groups of rats. Supplementary vanadium characteristically reduced the appearance of PNs of more than 3 mm in size in groups C, E and G as compared with group A. Mean nodular volume was found to be inhibited following vanadium supplementation as compared with group A, but a statistically significant ($P < 0.05$) result was obtained only with group C. There was a significant decrease ($P < 0.01$) in nodule volume as percentage of liver volume in group C as compared with group A though an insignificant decrease in this feature was observed in the other two vanadium-supplemented groups (i.e. groups E and G).

*Effect of vanadium on induction of GGT-positive foci*

While the livers of rats in normal group (group B) as well as vanadium control groups (i.e. groups D, F and H) were found to be normal in terms of histochemical observations, the GGT-positive foci developed in all DENA-treated groups. In groups C and E, vanadium supplementation significantly ($P < 0.001$ and 0.05 respectively) attenuated GGT-positive foci development (no. $cm^{-2}$) in comparison with group A (Table V). A significantly decreased ($P < 0.01$) GGT-positive focal area and percentage of liver parenchyma occupied by foci were also observed in group C as compared with group A. In the presence of vanadium, GGT-positive lesions remodelled to greater extents, as could be seen from the lack of uniformity of GGT histochemistry and irregular outlines (non-uniform foci) coupled with low labelling index (Table V). However, the results were found to be mostly pronounced and statistically significant in group C as compared with group A.

*Effect of vanadium on hepatic histology*

Phenotypically altered hepatocyte populations including PNs were found scattered in the livers of all DENA-treated groups (i.e. groups A, C, E and G) but no such alterations were noticeable in untreated normal controls (group B) or in vanadium controls (i.e. groups D, F and H) (data not shown). The H&E-stained sections of liver slices revealed focal changes that were clearly distinguishable from the surrounding normal parenchyma. In group A, a gross alteration in hepatocellular architecture was found and the hepatocytes appeared oval in shape. The altered hepatocytes of foci and nodules were found to be consistently enlarged with more than one nucleus, which were largely vesiculated. Some nuclei in the cells were large and hyperchromatic with prominent and centrally located nucleoli. Extensive vacuolation was observed in the cytoplasm around the nucleus with masses of acidophilic material. In contrast, the cellular architecture of hepatic lobules seemed to be almost like normal liver in group C, which received vanadium supplementation during the entire period of the study. Liver sections from this group presented only a few clear cell foci. The cells were generally filled with cytoplasmic material and were less vacuolated than group A. The size of the nuclei was essentially the same as that of normal cells, and cells with two nuclei were considerably fewer than in group A. In group E, i.e. the group which received vanadium only before initiation, a predominance of clear rather than acidophilic cell foci was seen. Cells with two nuclei were less common than in group A and the size of the nuclei appeared similar to that of normal cells. However, a moderate improvement in vacuolation and compactness of hepatocytes in group E was evident when compared with group A, but these improvements over group A were of lesser extent as compared with group C. Treatments of rats with vanadium during the promotional phase (group G) only marginally improved the hepatocellular phenotype from group A, as was revealed by histological examination (data not shown).

*Effect of vanadium on hepatic enzymatic activity of GGT*

The enzymatic activity of GGT in the cytosol of PNs induced by DENA alone (group A), as measured quantitatively, was more than 72-fold greater than that found in the liver of untreated controls (group B) and about 2.8-fold higher than in NNSP (Table VI). Vanadium supplementation was found to be effective in reducing the high activity of GGT in both the PNs and NNSP significantly in groups C and E and insignificantly in group G as compared with group A. Here also vanadium-mediated reduction in GGT activity was maximally observed in group C animals. On the other hand, no significant alteration in GGT activity was noticed in vanadium control rats (i.e. groups D, F and H) as compared with their normal counterparts (group B).

**Table IV** Effect of vanadium supplementation (0.5 p.p.m.) on the size distribution and growth of persistent nodules in the livers of rats initiated with DENA and promoted by PB

| Group | No. of rats | *Nodules relative to size (% of total no.)* $\geq 3$ mm | $<3->1$ mm | $\leq 1$ mm | Mean nodular volume[a] ($cm^3$) | Nodular volume/liver volume[b] (%) |
|---|---|---|---|---|---|---|
| A | 10 | 40.2 | 30.8 | 28.9 | $1.42 \pm 0.27^c$ | $68.4 \pm 6.3$ |
| C | 5 | 26.9 | 34.6 | 38.4 | $0.74 \pm 0.09^d$ | $40.2 \pm 4.8^e$ |
| E | 7 | 34.5 | 33.8 | 31.6 | $0.93 \pm 0.15$ | $53.2 \pm 5.2$ |
| G | 8 | 37.3 | 29.8 | 32.7 | $1.19 \pm 0.21$ | $60.1 \pm 5.8$ |

[a]Individual nodule volumes were calculated from two perpendicular diameters on each nodule. [b]One gram of liver was assumed to occupy 1 $cm^3$ for this calculation. [c]Each value represents the mean ± s.e. [d]$P < 0.05$ and [e]$P < 0.01$ as compared with group A.

**Table V** Influence of vanadium supplementation (0.5 p.p.m.) on the induction of GGT-positive liver cell foci in rats initiated with DENA followed by promotion with PB

| Group | No. of rats | No. of foci $cm^{-2}$ | Focal area ($mm^2$) | % area of liver parenchyma occupied by foci (%) | Non-uniform foci | Labelling index |
|---|---|---|---|---|---|---|
| A | 10 | $26.7 \pm 3.5^a$ | $0.48 \pm 0.06$ | $8.25 \pm 0.52$ | $11.31 \pm 2.11$ | $2.31 \pm 0.08$ |
| C | 12 | $7.3 \pm 0.6^b$ | $0.29 \pm 0.01^c$ | $6.12 \pm 0.32^c$ | $32.70 \pm 5.50^c$ | $1.62 \pm 0.06^b$ |
| E | 12 | $16.7 \pm 2.5^d$ | $0.34 \pm 0.07$ | $7.37 \pm 0.46$ | $18.78 \pm 3.10$ | $2.13 \pm 0.05$ |
| G | 11 | $20.1 \pm 3.0$ | $0.41 \pm 0.05$ | $7.92 \pm 0.56$ | $16.51 \pm 3.22$ | $2.21 \pm 0.07$ |

[a]Each value represents the mean ± s.e. [b]$P < 0.001$, [c]$P < 0.01$ and [d]$P < 0.05$ as compared with group A.

**Table VI** Alterations in the enzymatic activity of cytosolic GGT in the livers of rats of different experimental groups

| Group | GGT activity (nmol product formed $min^{-1}$ $mg^{-1}$ protein) PNs (n = 5) | NNSP (n = 5) | Control (n = 4) |
|---|---|---|---|
| A | 56.90 ± 7.21[a,b] | 20.38 ± 3.70[c] | 0.78 ± 0.10 |
| C | 12.15 ± 2.90[c,d] | 5.12 ± 1.11[c,e] | 0.83 ± 0.12 |
| E | 23.25 ± 4.12[b,e] | 9.31 ± 2.45[c,f] | 0.72 ± 0.09 |
| G | 37.81 ± 6.41[b] | 14.17 ± 3.92[g] | 0.87 ± 0.13 |

[a]Each value represents the mean ± s.e. [b]$P < 0.001$, [c]$P < 0.01$ and [g]$P < 0.02$ as compared with the corresponding control, i.e. groups B,D,F and H for groups A,C,E and G respectively. [d]$P < 0.001$, [e]$P < 0.01$ and [f]$P < 0.05$ as compared with group A.

### Discussion

The results of our present investigation clearly demonstrate that in this particular two-stage model of hepatocarcinogenesis in rats the supplementation of 0.5 p.p.m. vanadium during the entire experiment, before initiation and during promotion greatly reduced the incidence, multiplicity and size of visible PNs with a concurrent arrest in the number and spread of GGT-positive hepatic foci in total liver parenchyma. In the promotional event, however, these changes did not have any statistical significance. Our data, thus, reveal the unique protective role of vanadium against chemically induced liver tumorigenesis in rats and corroborate our previous findings (Bishayee and Chatterjee, 1995). This time the anticarcinogenic potential of vanadium is primarily observed on the initiation phase and only secondarily on the promotion stage. In this regard, it is interesting to note that continuous long-term exposure to a low dose of vanadium would elicit a greater protection in terms of the magnitude of preneoplasia than exposure at either the initiation or promotion phase alone.

In our experiment, the supplementation of 0.5 p.p.m. vanadium in drinking water, especially during the entire period of the study, resulted in fewer rats developing visible PNs and a smaller number of nodules per nodule-bearing rat liver than those observed in DENA control animals. Another striking observation of the study was the vanadium-mediated inhibition of the appearance of PNs more than 3 mm in size with a concurrent attenuation of nodular volume as well as nodular volume as a percentage of liver volume. Although it is evident that not all the hepatocyte nodules become cancerous during the lifespan of the animals, numerous observations support the concept that the nodules are the precursors of hepatic cancer (Farber, 1980; Williams, 1980). Moreover, there is a large body of observational experience in experimental and human disease correlating the number and size of nodular hyperplasia and hepatocarcinoma (Farber and Cameron, 1980; Farber, 1990). In view of this, inhibition of nodule growth and enhancement of their regression by vanadium as observed in our study may be important for cancer prevention, especially if one considers that the PNs are easily recognisable and have a low tendency to regress spontaneously. Again, the food and fluid intakes and changes in body weights among different experimental groups were found to be statistically similar. This feature is of paramount importance because nutritional deprivation causing body weight loss may parallel a decrease in tumour volume (Waitzberg *et al.*, 1989). Thus, the observed inhibitory effect of vanadium on nodule appearance and its growth is unlikely to be mediated through the impairment of nutritional status in the experimental animals.

It is generally accepted that GGT-positive foci appear to be the first discernible evidence for the occurrence of tumour initiation (Farber, 1980; Pitot and Sirica, 1980). Moreover, the use of GGT-positive foci in initiation–promotion bioassay is predicted on the assumption that the incidence of foci correlates with the eventual tumour yield that would have occurred had the assay continued until tumour formation (Farber, 1980; Pereira, 1982). In the present study, our results clearly showed an inhibitory role of vanadium on the number of GGT-positive preneoplastic focal lesions per $cm^2$ of the livers of rats initiated with DENA. As GGT-positive foci represent a transient step to malignancy (Tatematsu *et al.*, 1988), the ability of vanadium to reduce the development of GGT-positive foci suggests that this trace element can greatly affect the initiation stages of hepatocarcinogenesis by preventing the initiated cells from growing into preneoplastic foci through an alteration in the efficiency at which DENA can initiate foci appearance. The potential role of vanadium in reducing the number of foci per $cm^2$ of liver area was also reflected through a relatively high remodelling and low labelling index. This strongly indicates that a progressive loss of growth capacity by putative preneoplastic cells and their differentiation into normal-appearing hepatocytes proceed to a greater extent in the presence of vanadium.

According to the well-accepted hypothesis of Pitot *et al.* (1989), the number and size of altered liver cell foci indicate initiating and promoting activities respectively. In our study, vanadium supplementation not only decreased the number of GGT-positive preneoplastic foci but also caused a decrement in the focal area with a concomitant reduction in focal area as a percentage of liver area, though the results were statistically more significant with respect to focal number. However, this strongly points out the influence of vanadium in inhibiting or slowing the growth of altered liver cell foci. The observed effect of vanadium on focal growth may represent a selective toxicity to proliferating cells by virtue of the fact that they are proliferating compared with a relatively non-proliferating background and thereby eventually suppress the occurrence of hepatocarcinogenesis. In this regard, it is interesting to note that, although GGT-positive foci appeared in the livers of all the vanadium-treated rats concomitant with DENA administration (foci incidence 100%), only a few rats exhibited PNs in their livers (nodule incidence 41.6–72.7% in the three vanadium-supplemented groups). Since PNs arise from enzyme-altered focal growth (Feo *et al.*, 1988), our present findings could be explained in the light of the fact that, although the precursor lesions were still present in the livers of vanadium-exposed rats, their growth rate slowed to such an extent that appearance of PNs was delayed beyond the experimental end point owing to an increased latency period. This interpretation is supported by our histological assessment, in which the livers of vanadium-supplemented animals (specially in groups C and E) presented a well-maintained liver architecture with relatively less acidophilic hepatocyte areas than DENA controls.

The enzymatic activity of GGT has been identified as a possible positive marker for preneoplastic hepatocytes (Cameron *et al.*, 1978; Hanigan and Pitot, 1985). In the current study its activity was measured quantitatively in different cell populations during the induction of liver cancer with DENA in the presence or absence of vanadium. Although GGT activity is located inside the plasma membrane, we performed our study using cytosol as it is generally released in a soluble form by homogenisation. Elhkim *et al.* (1992) also observed that at least 80% of the total GGT activity was present in the cytosolic fraction. Further, PB is known to be a very weak inducer of GGT alone and, in combination with the initiating carcinogen DENA, the induction increases greatly (Shirai *et al.*, 1985). The exponential increase in the activity of GGT in PNs and NNSP following DENA injection as observed here resembled a growth process which originated as a response to toxic cellular injury. As there is evidence for a close connection between GGT activation and carcinogenesis (Fiala and Fiala, 1973), a large increase in this enzyme activity could be correlated with a high nodule incidence, a high total number and a large spread of nodules and foci in hepatic tissue. Vanadium-mediated inhibition of GGT-positive hepatocyte foci and PNs during rat liver carcinogenesis initiated with DENA and promoted by PB was well reflected in the relatively low level of this enzymatic activity, which was best observed in the group in which treatment with vanadium continued throughout the study. This might indicate a change in the plasma membrane of the cells that could be related to the ultimate development of neoplasia, since membrane changes are most easily related theoretically to neoplastic behaviour (Nicolson, 1976).

DENA and other nitrosamines are thought to confer their carcinogenic action through their metabolic activation, generating DNA-binding alkylating agents (Swenberg *et al.*, 1991). Thus, it is likely that the observed inhibitory effect of vanadium on rat liver carcinogenesis may be related to some modification of the metabolic activation and/or detoxification of the particular carcinogen employed. We previously observed that subchronic oral administration of vanadium at very low doses significantly elevates the activity of hepatic and intestinal glutathione *S*-transferase (GST) in rats (Bishayee and Chatterjee, 1993). GST is a well-known cytosolic phase II enzyme which adds functional groups to the carcinogen, thereby lowering its biological activity and increasing its excretability. Recently, the induction of GST enzyme activity has been suggested to be a protective mechanism of a number of naturally occurring dietary anticarcinogens (Tanaka, 1992; Wattenberg, 1992; Zheng *et al.*, 1992; Nijhoff *et al.*, 1993). The induction of GST by vanadium could lead to enhanced carcinogen elimination as well as a reduction in carcinogen–DNA adduct formation and subsequent expression of preneoplastic lesions and ultimately neoplasia. This may be one of the underlying biochemical mechanisms of the chemopreventive action of this dietary micronutrient. However, full appreciation of this needs further study.

Regardless of the mechanism, based on the results reported here, vanadium could be considered a potential cancer chemopreventive agent whose effect is presumably based on inhibition of growth of preneoplastic tissue, coupled with its remodelling to normal-appearing liver tissue. This attribute could be considered important, as this trace element may open new perspectives for the clinical therapy of malignancies in human subjects in whom exposure history, genetics or other predisposing events raise the probability of occurrence of cancer to an alarmingly high level.

### Acknowledgments

This work was supported by a research grant [No. 9/96(177)/91-EMR-I] from the Council of Scientific and Industrial Research (CSIR), Government of India. AB was the recipient of a CSIR Senior Research Fellowship during the study. We are indebted to Dr SN Kundu for histopathological evaluation of tissue samples and to Dr R Karmakar for assistance in focal analysis. We are also grateful to A Mandal for the histological preparation of samples.

### References

AQUINO TM, PORTA EA, SABLAN HM AND DORADO RD. (1985). Effects of selenium supplementation on hepatocarcinogenesis in rats. *Nutr. Cancer*, **7**, 25–35.

BISHAYEE A AND CHATTERJEE M. (1993). Selective enhancement of glutathione S-transferase activity in liver and extrahepatic tissues of rat following oral administration of vanadate. *Acta Physiol. Pharmacol. Bulg.*, **19**, 83–89.

BISHAYEE A AND CHATTERJEE M. (1994). Increased lipid peroxidation in tissues of the catfish *Clarias batrachus* following vanadium treatment: *in vivo* and *in vitro* evaluation. *J. Inorg. Biochem.*, **54**, 277–284.

BISHAYEE A AND CHATTERJEE M. (1995). Inhibition of altered liver cell foci and persistent nodule growth by vanadium during diethylnitrosamine-induced hepatocarcinogenesis in rats. *Anticancer Res.* (in press).

CAMERON R, KELLEN J, KOLIN A, MALKIN A AND FARBER E. (1978). γ-Glutamyltransferase in putative premalignant cell populations during hepatocarcinogenesis. *Cancer Res.*, **38**, 823–829.

CAMPBELL HA, PITOT HC, POTTER VR AND LAISHES BA. (1982). Application of quantitative stereology to the evaluation of enzyme altered foci in rat liver. *Cancer Res.*, **42**, 465–472.

CHAKRABORTY A AND CHATTERJEE M. (1994). Enhanced erythropoietin and suppression of gamma-glutamyltranspeptidase (GGT) activity in murine lymphoma following administration of vanadium. *Neoplasma*, **41**, 291–296.

COKER HAB, THOMAS AE AND AKINTONWA A. (1991). Determination of the total level of nitrosamines in select consumer products in Lagos area of Nigeria. *Bull. Environ. Contam. Toxicol.*, **47**, 706–710.

CRANS DC, BUNCH RL AND THEISEN LA. (1989). Interaction of trace levels of vanadium (IV) and vanadium (V) in biological systems. *J. Am. Chem. Soc.*, **111**, 7597–7607.

DJORDJEVIC C AND WAMPLER GL. (1985). Antitumor activity and toxicity of peroxo heteroligand vanadates (V) in relation to biochemistry of vanadium. *J. Inorg. Biochem.*, **25**, 51–55.

ELHKIM MO, DECLOITRE F, MARTIN M, LORIDON-ROSA B AND FRAYSSINET C. (1992). Role of diethylnitrosamine, 2-acetylaminofluorene and partial hepatectomy in the expression of glutathione-S-transferase-P and gamma-glutamyltranspeptidase in the early steps of rat liver carcinogenesis. *Tumor Biol.*, **13**, 152–161.

FARBER E. (1980). The sequential analysis of liver cancer induction. *Biochim. Biophys. Acta*, **605**, 149–166.

FARBER E. (1984*a*). The multistep nature of cancer development. *Cancer Res.*, **44**, 4217–4223.

FARBER E. (1984*b*). Cellular biochemistry of the stepwise development of cancer with chemicals. *Cancer Res.*, **44**, 5463–5474.

FARBER E. (1990). Clonal adaptation during carcinogenesis. *Biochem. Pharmacol.*, **39**, 1837–1846.

FARBER E AND CAMERON R. (1980). The sequential analysis of cancer development. *Adv. Cancer Res.*, **35**, 125–226.

FEO F, GARCEA R, DAINO L AND PASCALE R. (1988). Mechanisms of the inhibition of liver hepatocarcinogenesis promotion by *S*-adenosyl-L-methionine. In *Experimental Hepatocarcinogenesis*, Roberfroid MB and Preat V. (eds) pp. 197–207. Plenum: New York.

FIALA S AND FIALA ES. (1973). Activation by chemical carcinogens of gamma-glutamyltranspeptidase in rat and mouse liver. *J. Natl Cancer Inst.*, **51**, 151–158.

FRENCH RJ AND JONES PJ. (1993). Role of vanadium in nutrition: metabolism, essentiality and dietary consideration. *Life Sci.*, **52**, 339–346.

GARCEA R, DAINO L, PASCALE R, SIMILE MM, PUDDU M, FRASSETTO S, COZZOLINO P, SEDDAIU MA, GASPA L AND FEO F. (1989). Inhibition of promotion and persistent nodule growth by *S*-adenosyl-L-methionine in rat liver carcinogenesis: role of remodelling and apoptosis. *Cancer Res.*, **49**, 1850–1856.

GREENWALD P, CULLEN JW AND McKENNA JW (1987). Cancer prevention and control: from research through application. *J. Natl Cancer Inst.*, **79**, 389–400.

GULLAPALLI S, SHIVASWAMY V, RAMASARMA T AND RAMAKRISHNAKURIP CK. (1989). Redistribution of subcellular calcium in rat liver on administration of vanadate. *Mol. Cell. Biochem.*, **90**, 155–164.

HANAUSKE U, HANAUSKE A-R, MARSHALL MH, MUGGIA VA AND VON HOFF DD. (1987). Biphasic effect of vanadium salts on *in vitro* tumor colony growth. *Int. J. Cell Clon.*, **5**, 170–178.

HANIGAN MH AND PITOT HC. (1985). Gamma-glutamyltranspeptidase – its role in hepatocarcinogenesis. *Carcinogenesis*, **6**, 165–172.

KINGSNORTH AN, LAMURAGLIA GM, ROSS JS AND MALT RA. (1986). Vanadate supplements and 1,2-dimethylhydrazine induced colon cancer in mice: increased thymidine incorporation without enhanced carcinogenesis. *Br. J. Cancer*, **53**, 683–686.

KOPF-MAIER P. (1987). Cytostatic non-platinum metal complexes: new perspective for the treatment of cancer? *Naturwissenschaften*, **74**, 374–382.

LOWRY OH, ROSEBROUGH NJ, FARR AL AND RANDALL RL. (1951). Protein measurement with the Folin phenol reagent. *J. Biol. Chem.*, **193**, 265–275.

MARSMAN DS AND POPP JA. (1994). Biological potential of basophilic hepatocellular foci and hepatic adenoma induced by the peroxisome proliferator, Wy-14,643. *Carcinogenesis*, **15**, 111–117.

MORENO FS, RIZZI MBS, DAGLI MLZ AND PENTEADO MVC. (1991). Inhibitory effects of β-carotene on preneoplastic lesions induced in Wistar rats by the resistance hepatocyte model. *Carcinogenesis*, **12**, 1817–1822.

NECHAY BR, NINNINGA LB, NECHAY SE, POST RL, GRANTHAM JJ, MACARA IG, KUBENA LF, PHILLIPS TD AND NIELSEN FH. (1986). Role of vanadium in biology. *Fed. Proc.*, **45**, 123-132.
NICOLSON G. (1976). Trans-membrane control of the receptors on normal and malignancy. *Biochim. Biophys. Acta*, **458**, 1-71.
NIJHOFF WA, GROEN GM AND PETERS WHM. (1993). Induction of rat hepatic and intestinal glutathione S-transferases and glutathione by dietary naturally occurring anticarcinogens. *Int. J. Oncol.*, **3**, 1131-1139.
PEREIRA MA. (1982). Rat liver foci bioassay. *J. Am. Coll. Toxicol.*, **1**, 101-117.
PERERA MIR, KATYAL SL AND SHINOZUKA H. (1987). Choline deficient diet enhances the initiating and promoting effects of methapyrilene hydrochloride in rat liver as assayed by the induction of γ-glutamyltranspeptidase-positive hepatocyte foci. *Br. J. Cancer*, **56**, 774-778.
PITOT HC AND SIRICA AE. (1980). The stages of initiation and promotion in hepatocarcinogenesis. *Biochim. Biophys. Acta*, **605**, 191-215.
PITOT HC, CAMPBELL HA AND MARONPOT R. (1989). Critical parameters in the quantitation of the stages of initiation, promotion and progression in one model of hepatocarcinogenesis in the rat. *Toxicol. Pathol.*, **17**, 594-605.
RUTENBERG AM, KIM H, FISCHBEIN JW, HANKER JS, WASSERKRUG HL AND SELIGMAN AM. (1969). Histochemical and ultrastructural demonstration of gamma-glutamyltranspeptidase activity. *J. Histochem. Cytochem.*, **17**, 517-526.
SABBIONI E, POZZI G, PINTER A, CASELLA L AND GARATTINI S. (1991). Cellular retention, cytotoxicity and morphological transformation by vanadium (IV) and vanadium (V) in BALB 3T3 cell lines. *Carcinogenesis*, **12**, 47-52.
SABBIONI E, POZZI G, DEVOS S, PINTAR A, CASELLA L AND FISCHBACH M. (1993). The intensity of vanadium (V)-induced cytotoxicity and morphological transformation in BALB/3T3 cells is dependent on glutathione-mediated bioreduction to vanadium (IV). *Carcinogenesis*, **14**, 2565-2568.
SARDAR S, GHOSH R, MONDAL A AND CHATTERJEE M. (1993). Protective role of vanadium in the survival of hosts during the growth of a transplantable murine lymphoma and its profound effects on the rates and patterns of biotransformation. *Neoplasma*, **40**, 27-30.
SARKAR A, MUKHERJEE B AND CHATTERJEE M. (1994). Inhibitory effect of β-carotene on chronic 2-acetylaminofluorene induced hepatocarcinogenesis in rat: reflection in hepatic drug metabolism. *Carcinogenesis*, **15**, 1055-1060.
SCHERER E AND EMMELOT P. (1976). Kinetics of induction and growth of enzyme-deficient islands involved in hepatocarcinogenesis. *Cancer Res.*, **36**, 2544-2554.
SERFONTEIN WJ AND HURTER P. (1966). Nitrosamines as environmental carcinogens II. Evidence for the presence of nitrosamines in tobacco smoke condensate. *Cancer Res.*, **26**, 575-579.
SHIRAI T, IMAIDA K, OHSHIMA M, FUKUSHIMA S, LEE M-S, KING CM AND ITO N. (1985). Different responses to phenobarbital promotion in the development of γ-glutamyltranspeptidase-positive foci in the liver of rats initiated with diethylnitrosamine, N-hydroxy-2-acetylaminofluorene and aflatoxin $B_1$. *Jpn J. Cancer Res.*, **76**, 16-19.
SWENBERG JA, HOEL DG AND MAGEE PN. (1991). Mechanistic and statistical insight into the large carcinogenesis bioassays on N-nitrosodiethylamine and N-nitrosodimethylamine. *Cancer Res.*, **51**, 6409-6414.
TANAKA T. (1992). Cancer chemoprevention. *Cancer J.*, **5**, 11-16.
TATE SS AND MEISTER A. (1974). Interaction of γ-glutamyltranspeptidase with amino acids, dipeptides and derivatives and analogs of glutathione. *J. Biol. Chem.*, **249**, 7593-7602.
TATEMATSU M, NAGAMINE Y AND FARBER E. (1983). Redifferentiation as a basis for remodelling of carcinogen-induced hepatocyte nodules to normal appearing liver. *Cancer Res.*, **43**, 5049-5058.
TATEMATSU M, MERA Y, INOUE T, SATOH K, SATO K AND ITO N. (1988). Stable phenotypic expression of glutathione-S-transferase placental type and unstable phenotypic expression of γ-glutamyltranspeptidase in rat liver preneoplastic and neoplastic lesions. *Carcinogenesis*, **9**, 215-220.
THOMPSON HJ, CHASTEEN ND AND MEEKER LD. (1984). Dietary vanadyl (IV) sulfate inhibits chemically-induced mammary carcinogenesis. *Carcinogenesis*, **5**, 849-851.
WAITZBERG DL, GONCALVES EL, FAINTUCH J, BEVILACOUA LR, ROCHA CL AND CDOGNI AM. (1989). Effects of diets with different protein levels on the growth of Walker 256 carcinosarcoma in rats. *Brazil J. Med. Biol. Res.*, **22**, 447-455.
WATTENBERG LW. (1985). Chemoprevention of cancer. *Cancer Res.*, **48**, 1-8.
WATTENBERG LW. (1992). Inhibition of carcinogenesis by minor dietary constituents. *Cancer Res.*, **52**, 2085-2091.
WILLIAMS GM. (1980). The pathogenesis of rat liver cancer caused by chemical carcinogenesis. *Biochim. Biophys. Acta*, **605**, 167-189.
WILLIAMS GM. (1989). The significance of chemically-induced hepatocellular altered foci in rat liver and application to carcinogen detection. *Toxicol. Pathol.*, **17**, 663-680.
YOSHIJI H, NAKAE D, KINUGASA T, MATSUZAKI M, DENDA A, TSUJII T AND KONISHI Y. (1991). Inhibitory effect of dietary iron deficiency on the induction of putative preneoplastic foci in rat liver initiated with diethylnitrosamine and promoted by phenobarbital. *Br. J. Cancer*, **64**, 839-842.
ZHENG G-Q, KENNEY P AND LAM LKT. (1992). Myristin: a potential cancer chemopreventive agent from parsley leaf oil. *J. Agric. Food Chem.*, **40**, 107-110.